# A Local Epidemic of Laced Heroin Causing Skin Necrosis

**DOI:** 10.7759/cureus.4782

**Published:** 2019-05-30

**Authors:** Jessica Houck, Latha Ganti

**Affiliations:** 1 Emergency Medicine, University of Central Florida College of Medicine / Hospital Corporation of America Graduate Medical Education (HCA GME) Consortium, Kissimmee, USA; 2 Emergency Medicine, Envision Physician Services, Orlando, USA

**Keywords:** heroin addiction

## Abstract

The authors present a case of severe skin necrosis resulting from injecting heroin laced with a substance known as "Rizzy" powder. This powder is a toxic substance used to color the petals of plants. This report reviews how to manage the complications of such adulterated heroin.

## Introduction

Heroin use in the United States is on the rise, with the U.S. Centers for Disease Control and Prevention (CDC) reporting that more than half a million Americans used heroin in 2013, representing a nearly three-fold increase since 2007 [1].

Heroin is a schedule 1 (class 1) drug. Drugs in this class are illegal because they have high abuse potential, no medical use, and severe safety concerns. One of the added risks of street heroin is that it contains an estimated 7%-10% to a maximum of 20% pure heroin with the remainder comprising filler substances [2]. Due to its illegal nature, it is often “cut” with other substances. The most common substances used to cut heroin include baking soda, sugar, starch, crushed over-the-counter painkillers, talcum powder, powdered milk, laundry detergent, caffeine, and rat poison [3].

Another common but deadly substance used in laced heroin is fentanyl, a potent synthetic opioid. The United States Drug Enforcement Agency reported a >300% increase in the laboratory testing of confiscated heroin from 2014 to 2015, with a 73% increase in deaths caused by synthetic opioids [4]. More recently, the CDC has confirmed laced marijuana with Brodifacoum (BDF), otherwise known as superwarfarin, to be the cause in at least 150 patients that presented with bleeding across 11 states since March 2018. This resulted in eight reported fatalities [5-7].

The effects on the patient depend on which substance the heroin was cut with and how the heroin is taken. In this case, the authors describe a case of heroin cut with Rizzy powder, which results in disfiguring necrotic skin lesions.

## Case presentation

A 26-year-old female presented to our emergency department (ED) with the chief complaint of “sores on arms.” The patient was resting comfortably on the stretcher in a long sleeve jacket. She was a pleasant young woman who explained that she was an intravenous (IV) heroin drug user and that earlier that day, she had tried to check into a rehabilitation facility and was turned away. She reported that they would not take her until she sought medical attention for the “sores on her arms.”

She reported that she noticed the rash a few months ago, and, initially, it started as small spots but had gradually gotten worse each time she injected heroin. She also reported that her friends, who also used intravenous (IV) heroin, have similar lesions. She denied any pain or itchiness associated with the rash. She denied any fever, chills, or other symptoms associated with the rash.

**Figure 1:**
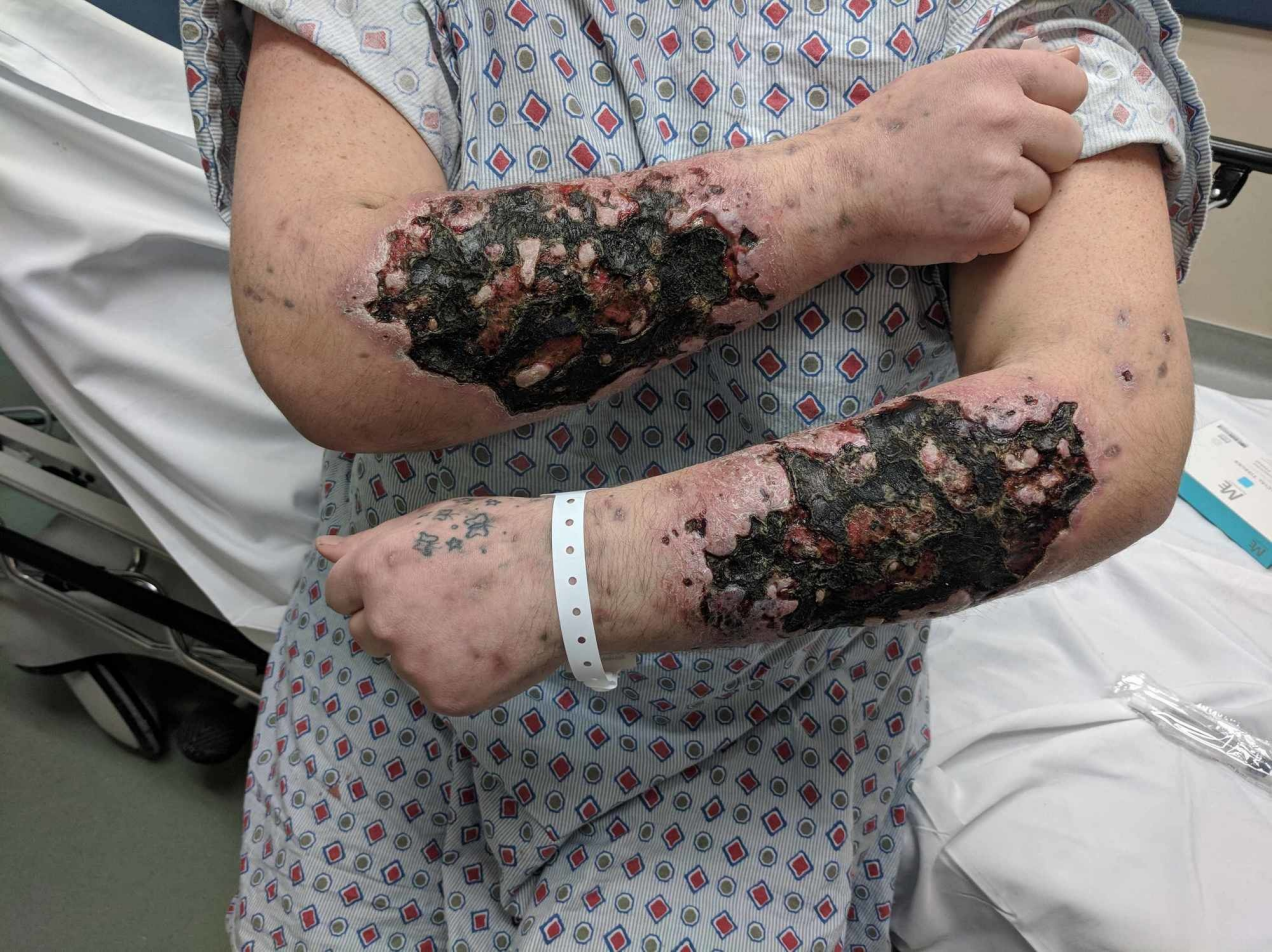
Black and green necrotic and scaly lesions of the patient's forearms.

Upon removing her jacket for the physical exam, it was noted that her forearms were necrotic with large green and black scales that felt like the texture of leather (Figure 1). There was no sensation in the areas affected, with distal preservation of neurovascular function. The remainder of her physical exam, including vital signs, was normal.

**Figure 2:**
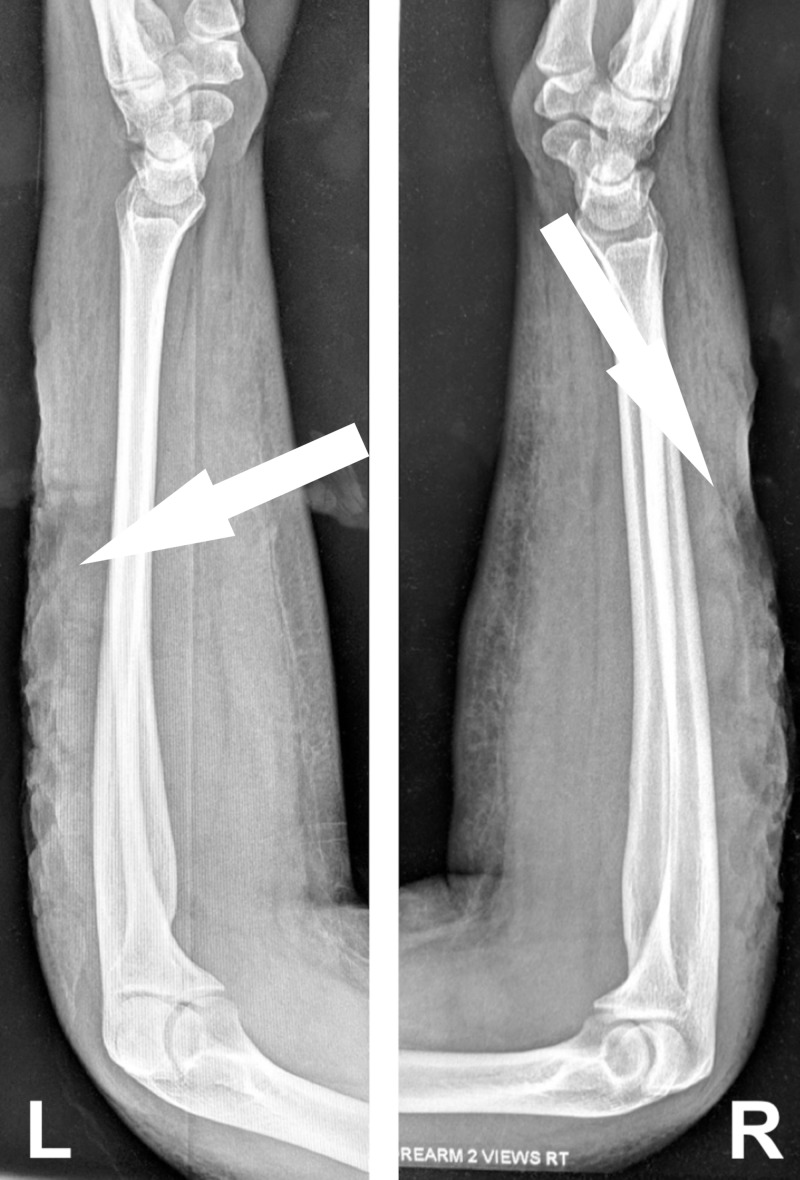
Radiographs of the forearms demonstrating gas within the soft tissue.

A full sepsis workup was initiated, including broad-spectrum antibiotics. Remarkably, her labs were all normal. Radiographs revealed gas in the subcutaneous tissue (Figure 2). The patient was admitted to the hospital and went for surgical debridement the next day. She did well post-op and was discharged home from the hospital.

## Discussion

Initially, it was thought that this patient was unintentionally using a synthetic opioid, which is well-known in Russia, called desomorphine and is also known as “krokodil” or “the flesh-eating zombie drug.” However, a few weeks after seeing this patient, a public service announcement was released by the sheriff’s department titled “Rizzy Powder” [8]. The safety bulletin addressed a trend of recently arrested IV heroin users in that were found to have necrotic skin lesions secondary to using heroin that had been cut with a powder called Rizzy, a powder used to dye flowers for centerpieces, which, once injected, causes skin necrosis. Rizzy is a concentrate used to keep flowers fresher for longer, with packaging that specifically says “Toxic. Do not ingest. Keep out of reach of children. Call Doctor immediately if ingested.”

As highlighted in the introduction section, Rizzy is not the first substance to be used to lace street drugs and will not be the last. For this reason, it is imperative to have a high index of suspicion in patients with a history of drug abuse.

## Conclusions

The differential diagnosis of skin manifestations in IV drug users should include both infection and the direct caustic effects of agents that may have been used to “cut” heroin by drug dealers. Sepsis work-up with broad-spectrum antibiotics should be initiated at the time of evaluation. Depending on the extent of skin involvement, patients may need to be transferred to a burn unit for possible skin grafting.
